# Compact Tactile Representations in Texture Classification

**DOI:** 10.3390/s26217044

**Published:** 2026-11-07

**Authors:** Frederico Luiz Martins de Sousa, Aref Sayareh, Mateus Nazario Coelho, Mateus Coelho Silva, Thiago Eustaquio Alves de Oliveira, Amilcar Soares Junior, Vinicius Prado da Fonseca

**Affiliations:** 1 Department of Computer Science, Memorial University of Newfoundland, St. John’s, NL A1C 5S7, Canada; 2 Center for Mathematics, Computation and Cognition, Federal University of ABC, Santo André 09280-560, SP, Brazil; 3 Department of Computer Science, Lakehead University, Orillia, ON L3V 0B9, Canada; 4 Department of Engineering Mathematics and Technology, University of Bristol, Bristol BS8 1TW, UK; 5 Department of Computer Science and Media Technology, Linnaeus University, 351 95 Växjö, Sweden

**Keywords:** signal fusion, tactile perception, texture classification, PCA, ICA, robotic manipulation

## Abstract

Robotic tactile perception benefits from short interactions and compact feature representations. This study investigates whether nine-channel magnetic, angular-rate, and gravity (MARG) signals can be fused for texture classification using shortened segments of a 12 s exploration. A dataset of 12 textures and 100 trials per texture was evaluated using principal component analysis (PCA), independent component analysis (ICA), and channel-wise temporal averaging (AVG), with segment lengths corresponding to nominal durations of 3, 6, and 9 s and four classifiers. All learned pipeline stages were fitted exclusively on training experiments within five-fold experiment-level cross-validation repeated with four predefined seeds. For the shortest evaluated segments (nominally 3 s), the best observed cross-validation (CV) estimates, averaged across four seeds for AVG, PCA, and ICA, were 99.41%, 99.09%, and 98.92%, respectively. At 9 s, the configurations selected by the highest observed CV means yielded for PCA, AVG, and ICA configurations that achieved 99.99%, 99.97%, and 99.98% with 126, 162, and 162 classifier input features, respectively. Separately, a compact PCA–Random Forest (RF) Pareto configuration achieved 99.99% with only 36 features. These results show that accurate texture classification is possible with substantially shorter tactile interactions under the evaluated protocol: AVG performs strongly at 3 s, whereas PCA offers compact representations at longer durations.

## 1. Introduction

Robots in unstructured environments face significant challenges due to the variability and unpredictability of the surroundings. Unlike structured environments, which are controlled and consistent, unstructured environments present diverse objects, surfaces, and obstacles that robots must work with. These conditions can hinder the robot’s ability to perform tasks reliably, as visual sensors alone often cannot provide sufficient information for accurate decision-making. For example, cameras may misclassify objects when visual cues are ambiguous, or they may fail entirely in situations with poor lighting, cluttered scenes, or occlusion [1].

To overcome these limitations, robots can complement vision with touch, allowing them to capture physical properties such as friction and roughness that are not accessible through vision alone. However, tactile sensing produces large volumes of high-dimensional and temporal data, raising the challenge of efficiently processing this information. In this context, sensor fusion becomes essential to integrate and reduce tactile signals while preserving the discriminative features needed for reliable texture classification.

As a reliable source of information for manipulation tasks, tactile sensors have been widely employed to address different challenges in robotics. Through physical interaction, tactile sensing also captures intrinsic object properties such as friction and surface roughness. For example, capacitive [2,3] and magnetic [4,5] sensors are commonly used to recognize object textures or assist with grasping. Lima et al. [6] investigated this by employing magnetic, angular-rate, and gravity sensors with nine degrees of freedom, together with a barometer to measure pressure, in an experiment aimed at texture classification.

Recent work has investigated learned multimodal fusion for tactile texture recognition. Chen et al. [7] proposed MFT-Net, combining convolutional feature extraction, channel attention, and Transformer-based temporal modeling to integrate force and acoustic signals. Their study evaluated the contributions of individual modalities, architectural components, and fusion strategies.

He et al. [8] reviewed bioinspired vision–tactile fusion systems, datasets, and applications, while Wang et al. [9] surveyed flexible tactile sensors and intelligent algorithms for signal interpretation. Providing a concrete example of complementary sensing, Liu et al. [10] integrated visual information with pressure and temperature measurements for object recognition under degraded visual conditions.

More closely related to compact tactile representations, Al et al. [11] combined pressure, accelerometer, and gyroscope signals in a soft robotic finger, applying principal component analysis (PCA) before convolutional neural network–long short-term memory (CNN-LSTM) texture classification from 2 s windows. Zhao et al. [12] selected nine of 386 descriptors for tactile shape recognition, while Huang et al. [13] combined friction and vibration features with PCA for food-graininess assessment.

These studies motivate a controlled assessment of how temporal context and multichannel representation affect tactile texture classification. In this paper, we examine a windowed representation that projects flattened multichannel windows onto PCA or independent component analysis (ICA) components and concatenates successive projections to preserve their temporal order. Our contribution is a systematic characterization of the trade-offs among observation length, classifier input dimensionality, and classification accuracy, supported by comparisons with channel-wise temporal averaging, raw multichannel representations, and reduced sensor groups.

Previous studies have examined how signal representation affects tactile classification. Wang et al. [14] classified seven fabric textures using a finger-shaped multi-channel capacitive tactile sensor. Their approach first transforms the tactile time series into the frequency domain via Fourier transform (FT), resamples the resulting spectra into a fixed size, and then applies incremental principal component analysis (IPCA) to compress them into a low-dimensional vector of at most seven dimensions. A k-nearest neighbors (KNN) classifier trained on these features achieved an accuracy of 90% and 96% on seven fabrics. Their results demonstrate the potential of compact tactile representations, and the main contribution of the current paper is the use of machine learning methods to achieve better results with compact tactile representations.

Najib and Cretu [15] classified twelve textures using sound and vibro-tactile data from the VicTac-12 dataset. Their approach combines statistical features (mean, standard deviation, median absolute deviation, and perceptual measure) with frequency-based features obtained via fast Fourier transform (FFT) and applies PCA to reduce the feature set to a compact representation. Multiple classifiers were compared, and the extreme gradient boosting random forest (XGBRF) model offered the best performance and computation time balance, achieving an F1-score of 0.91, while KNN represented the second best option. Their results demonstrate that the choice of sampling period has a significant impact on classification accuracy, while longer periods (2 s vs. 1 s) can improve performance at the cost of increased evaluation time, reinforcing the motivation to seek compact representations that preserve classification performance. Motivated by this use of compact data, our study examines PCA and ICA projections of multichannel time-series windows, evaluating the trade-offs among observation duration and classification accuracy.

In Lima et al. [6], the authors explored the BioIn-Tacto [16] tactile sensor for robotic texture categorization, achieving high classification accuracy with machine learning methods. The bio-inspired sensor uses magnetic, angular-rate, and gravity (MARG) and barometer modules to emulate human tactile sensing. BioIn-Tacto has also supported tactile perception tasks using multimodal temporal signals, including texture recognition on uneven surfaces and contact-guided surface perception, demonstrating its ability to encode contact information from external objects and textures.

Their work serves as a baseline for our efforts to improve classification through signal reduction and fusion. Their study considered 12 textures, with a tactile finger moving across each surface in a square-like path 100 times [17]. Each experiment lasted 12 s, during which 10 measurements were recorded at each step. Lima et al. [6] classified the textures using the full 12 s of a single signal among the 10 available measurements.

Building on the BioIn-Tacto texture dataset [17], this study investigates how multichannel representation and temporal context affect texture classification from shortened recording segments. The proposed pipeline divides each segment into non-overlapping multichannel windows, projects each flattened window into PCA or independent component analysis (ICA) components, and concatenates successive outputs to form a compact representation that retains their temporal order. We compare these representations with channel-wise temporal averaging across segment lengths, fusion-window sizes, retained component counts, and four classifiers. The main configuration study uses repeated experiment-level cross-validation, with scaling and learned transformations fitted exclusively on training experiments.

The contribution is a systematic characterization of the trade-offs among observation length, classifier input dimensionality, and classification accuracy, supported by complementary baseline comparisons and sensor-group ablations. Matched holdout comparisons assess whether the windowed PCA representation improves classification relative to raw multichannel concatenation while reducing feature count. Separately, sensor-group ablations examine whether accurate classification requires all nine MARG channels or can be retained using reduced sensor subsets. Together, these analyses identify compact operating configurations and characterize sensor-group redundancy under the evaluated acquisition condition. The results show that channel-wise temporal averaging is a strong comparator for the shortest evaluated segments, while PCA provides compact representations at longer segment lengths. Accelerometer-only and magnetometer-only PCA representations also retain high observed accuracy, indicating that combining all sensor groups does not consistently improve classification in this setting.

The rest of this paper is organized as follows: Section 2 describes the dataset, preprocessing, signal representations, and evaluation protocols. Section 3 presents the classification results, baseline comparisons, and sensor-group ablations. Section 4 discusses the findings, limitations, and directions for future research.

## 2. Materials and Methods

**Figure 1:**
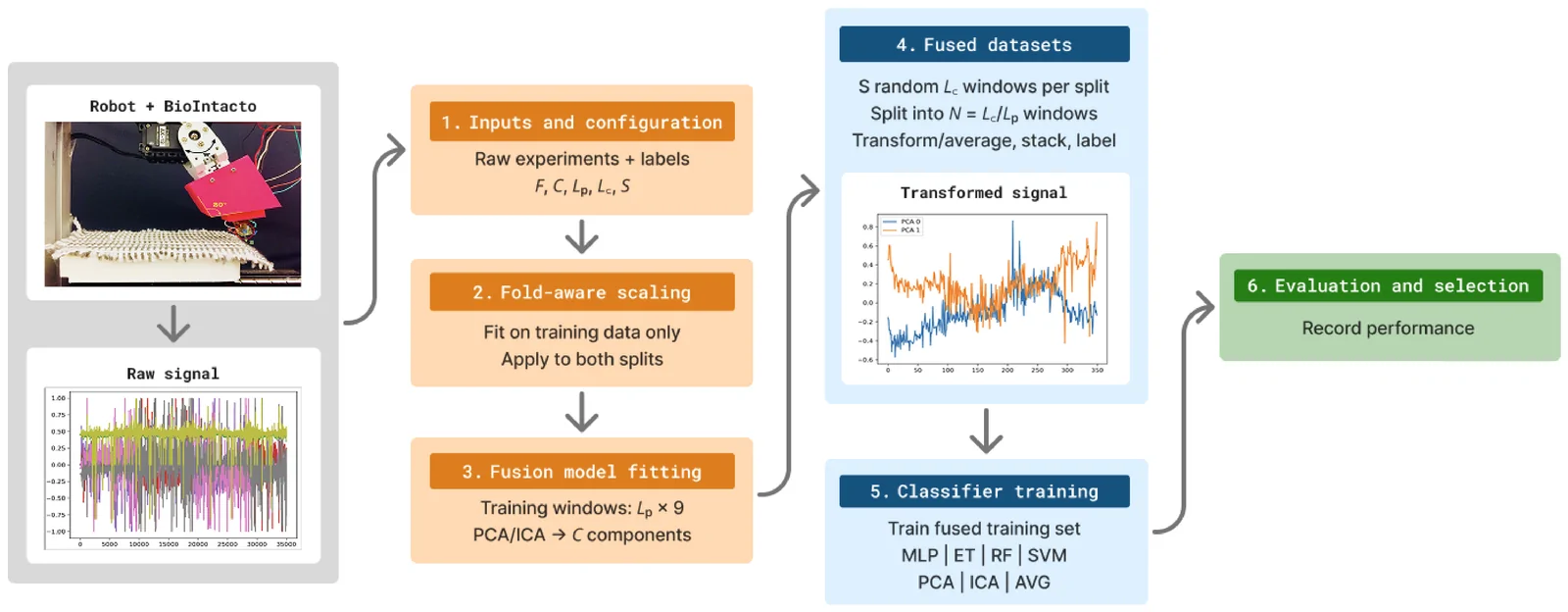
Overview of the fold-aware signal-fusion and texture-classification pipeline. The nine-channel tactile MARG signals are partitioned at the experiment level using five-fold cross-validation. For each fold and pipeline configuration $\left(F,C,L_{p},L_{c},S\right)$, min–max scaling and the PCA or ICA fusion model are fitted exclusively on the training experiments and subsequently applied to both training and validation data. Each training experiment contributes *S* randomly positioned segments and each validation experiment contributes 100 segments, all of duration $L_{c}$. Each segment is divided into $N=f_{s}L_{c}/L_{p}$ non-overlapping windows of $L_{p}$ samples, where $f_{s}=3000$ samples/s is the nominal reference rate used for segment extraction. Each window is represented by *C* components (for PCA/ICA) or by nine channel-wise temporal averaging (AVG) instances, and the resulting window representations are stacked and labeled. Multilayer perceptron (MLP), extra tree (ET), random forest (RF), and support vector machine (SVM) classifiers are trained on the fused training samples and evaluated on the corresponding validation samples. Performance is averaged across folds within each seed and then across four seeds to compare configurations.

The present study introduces a tactile-sensor fusion framework that transforms nine-channel MARG signals into compact representations for texture classification. PCA and ICA are evaluated alongside channel-wise temporal averaging using four machine learning classifiers. Figure 1 summarizes the fold-aware pipeline, including dataset construction, fusion-model fitting, classifier training, and performance evaluation. Table 1 also describes the variables and their values used in our methodology data pipeline.

### 2.1. Dataset Preparation and Random Extraction

As mentioned in Section 1, the proposed method was built using the previously published Multimodal Tactile Texture Dataset introduced by  Lima et al. [17], while also using their method as a baseline [6]. No new tactile recordings were collected for the present study. The acquisition setup and exploration protocol described below belong to the original dataset. Our contribution concerns the preparation, representation, and evaluation of those existing recordings. The previous classification study [6] is discussed separately as a reference for comparison. Furthermore, in that study, the authors use all texture exploration velocities (30 mm/s, 35 mm/s, and 40 mm/s) within the published dataset, while in our work, we will focus only on 30 mm/s.

The dataset was generated by one robotic hand with tactile sensors that sensed 12 different textures in a square path 100 times. The square path was explored for 12 s each time, and MARG and barometer signals were recorded. Therefore, the dataset contains 10 signals (or axes), 9 corresponding to the MARG data, and 1 to the barometer sensor for each touch exploration.

The dataset publication reports acquisition rates of 1500 Hz for the MARG signals and 350 Hz for the barometer. These reported acquisition rates should be distinguished from the frequency of timestamped entries in the released pickle files. Inspection of the local 30 mm/s MARG subset gives a median stored-record rate of approximately 3.14 k entries/s with variations. In the present study, the released MARG values were used in their existing row order, without temporal interpolation, resampling, or duplication of observations. Conversion to NumPy arrays preserved the retained signal values but did not retain the timestamp index. The acquisition rates reported by the dataset authors are therefore not interpreted as the rate of stored entries used by our implementation.

The implementation extracts segments containing 9000, 18,000, or 27,000 stored rows. These lengths were originally assigned nominal durations of 3, 6, and 9 s using a reference conversion of 3000 rows/s due to the frequency variation in the original dataset. The reference conversion is an implementation approximation, not an independently verified sensor acquisition rate. We therefore distinguish stored-row segment lengths from physical elapsed time and characterize their elapsed durations using the original timestamps. The barometer was excluded to keep the analysis focused on the nine MARG channels. Pressure measurements introduce an additional sensing modality with a different physical interpretation.

To avoid data leakage, all preprocessing steps that learn from the data are performed independently inside each cross-validation fold. For each fold, the normalization scaler is fitted only on the training experiments and then applied to both the training and validation experiments. Likewise, the fusion module, such as PCA or ICA, is fitted only using windows extracted from the training experiments of that fold. The validation experiments are only transformed and evaluated, ensuring that no information from the validation fold influences normalization, feature fusion, or classifier training.

In addition, we reduce the exploration time while fusing the signals. Therefore, if we want to select a shorter duration than 12 s from an experiment as a new sample, we can pick it from different parts of each experiment. In other words, this means that we can have multiple sub-experiments (shorter duration) in each original experiment. So, to create these sub-experiments from each experiment, we generate random extraction points, which indicate our sub-experiment starting point, and we pick the specific window frame from those starting points to apply our methods to them.

### 2.2. Signal Fusion

**Figure 2:**
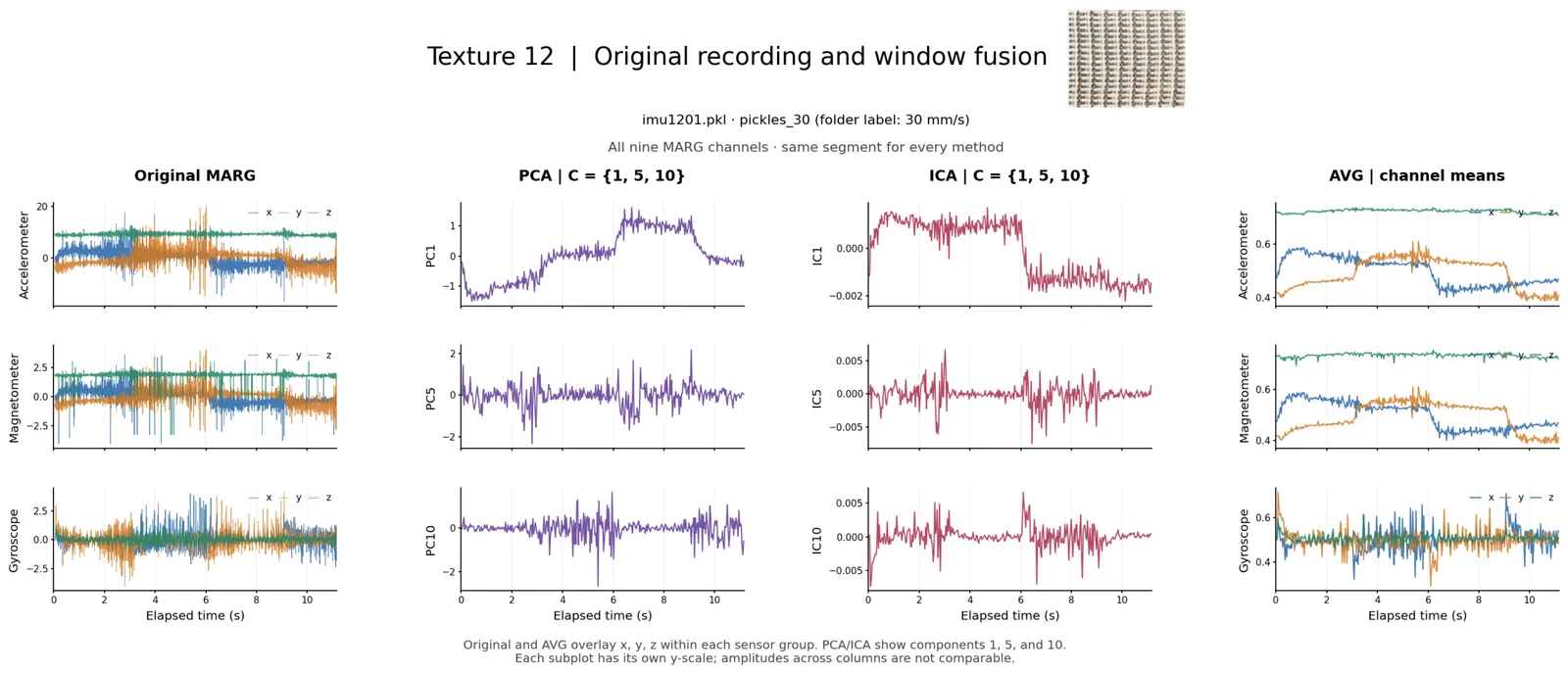
Texture 12 raw signals and transformations from PCA/ICA and dimensionality reduction for AVG methods.

Our study extracts random samples from each experiment, where PCA, ICA, and channel-wise temporal averaging (AVG) were selected to compare three approaches to constructing compact representations of multichannel tactile windows: variance-based linear projection, independence-based linear projection, and a fixed channel-wise temporal summary. This comparison examines whether learning joint combinations of channel and temporal coordinates provides useful classification representations relative to simple temporal summaries. Figure 2 shows texture 12 signals processed and transformed through PCA/ICA and AVG methods.

The PCA usage is motivated by the possibility that measurements from different MARG channels and neighboring positions within a window contain redundant variation associated with the same tactile interaction. It identifies orthogonal directions that successively maximize the variance represented in the training windows [18]. Its usefulness for classification depends on whether discriminative information is retained within a low-dimensional linear subspace. However, high variance does not necessarily imply texture relevance, because exploratory motion, orientation, and other acquisition effects may also contribute to the dominant components.

ICA provides a complementary criterion by seeking statistically independent, non-Gaussian latent components [19]. Its conventional source-separation interpretation assumes linear mixing of independent sources, with at most one Gaussian source. In this study, these assumptions are treated as a modeling hypothesis for the flattened tactile windows rather than established properties of the physical sensing processes. ICA components are therefore evaluated as learned classification features and are not interpreted as identified physical sources. Because ICA does not inherently order its components by importance, the component-subset comparisons use the training-based reconstruction-variance ranking described in Section 2.4.

Finally, AVG provides a simple comparator that requires no representation-fitting step. Each window is averaged independently over time for each channel, producing nine values without mixing channels. Concatenating successive summaries preserves their order across windows while discarding fluctuations within each window. AVG therefore tests whether coarse channel-level information is sufficient for classification under the evaluated acquisition condition.

We used non-overlapping windows on the signals to analyze them. A window frame is a small chunk of an experiment that is used in the fusion model for the transformation. The window frame used for the data fusion has different lengths in our experiment, described by the variable $L_{p}$ below. The implementation retains the first 36,000 measurements from each original exploration over the nine MARG channels, giving an input array of shape 36,000 × 9, while the shape of a fusion window frame with a length of $L_{p}$ (500 or 1500) is $\left(L_{p}\times 9\right)$, where we divide the original signal into an $L_{p}$ signal window.

**Figure 3:**
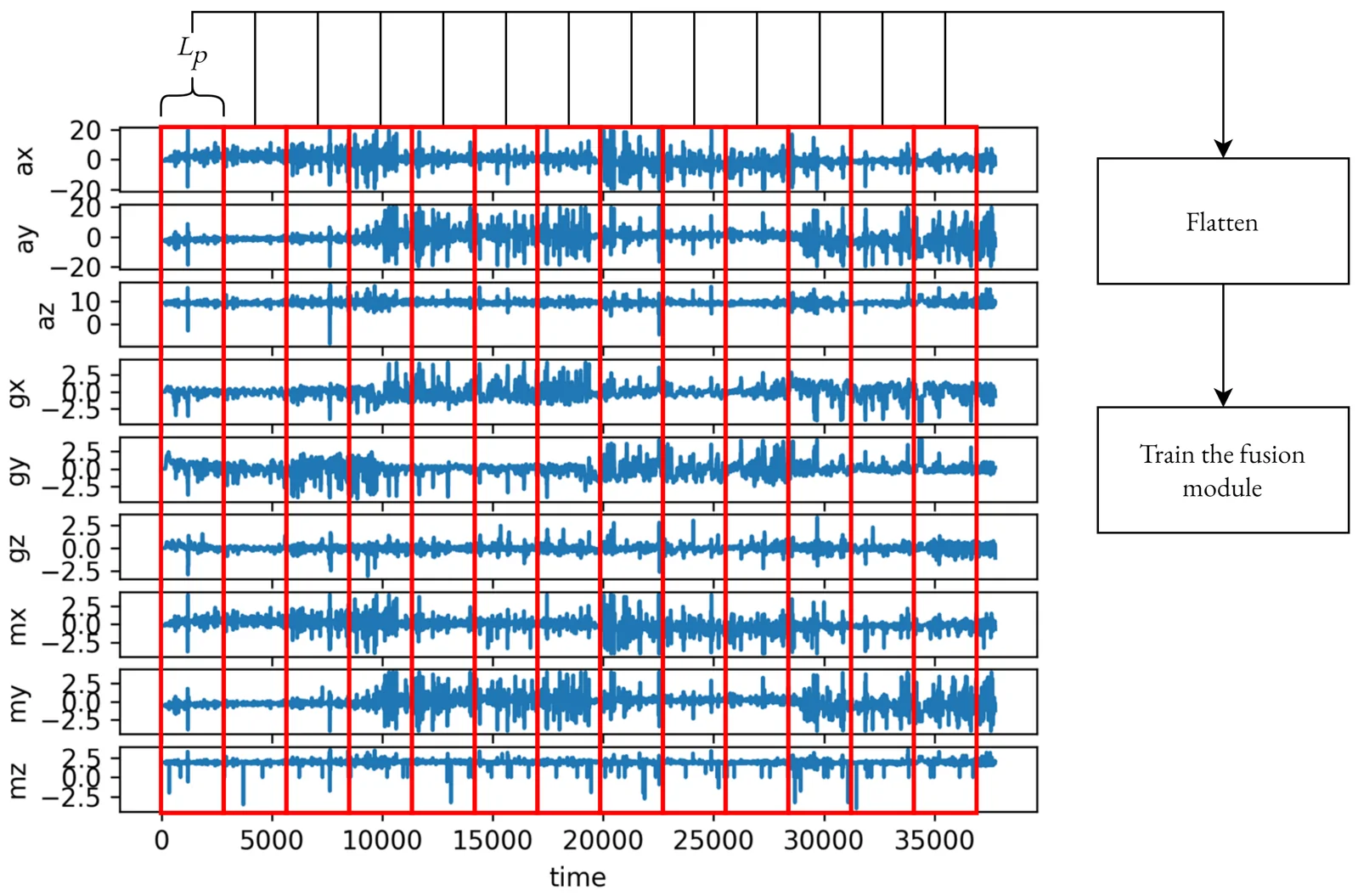
Splitting an experiment to windows frames to train the fusion module (e.g., PCA).

Therefore, for a given $L_{p}$, each window frame contains $L_{p}\times 9$ values, corresponding to $L_{p}$ time samples from the nine sensor signals. For PCA and ICA fusion, the window frame is flattened into a $9L_{p}$-dimensional vector and fitted onto *C* components, where *C* is experiment-defined and satisfies $C\leq 9\times L_{p}$, as Figure 3 displays. In contrast, AVG fusion averages the window over time and outputs one value per signal, resulting in a fixed 9-dimensional representation. Therefore, AVG is not fusion, being only a temporal average separately for each channel, retaining nine values per window.

Flattening assigns each channel–time position within a window to a fixed feature coordinate. This rearrangement preserves all recorded values and their positional correspondence; dimensionality reduction occurs during the subsequent projection. PCA and ICA can therefore combine information across both channels and temporal positions. However, neither method explicitly enforces temporal locality, extracts predefined frequency bands, or guarantees invariance to shifts within a window. The projection may discard discriminative oscillations or become sensitive to their position within the window. Concatenating successive window representations preserves their coarse temporal order but cannot recover information discarded within each window.

This design was adopted to investigate whether texture classification benefits from different temporal resolutions of tactile exploration. The window length $L_{p}$ controls the amount of temporal context provided to the fusion model: smaller windows emphasize short-term signal variations, whereas larger windows aggregate information over longer exploratory segments. At the same time, the fusion module avoids passing the full $9L_{p}$-dimensional raw window to the classifier. Instead, PCA and ICA transform the flattened window into *C* components, and AVG produces a fixed 9-dimensional summary. Therefore, the methodology evaluates both the effect of temporal window size and the effect of feature-space reduction on classification performance.

The training dataset was divided into window frames with lengths of $L_{p}$, then flattened into a 1D array and later used as samples to train the fusion module. Figure 3 illustrates this by showing the MARG signals of an experiment divided into the $L_{p}$ windows (red windows). Each of these windows contains a $\left(L_{p},9\right)$ data matrix that is flattened into one dimension of shape $1,L_{p}*9$ as a training sample and fed to the fusion modules to train them.

Because a single fusion window of length $L_{p}$ may contain limited temporal information, using only one transformed window may not provide enough information for the classifier. Conversely, increasing $L_{p}$ makes each fusion window longer, but reduces the number of temporal segments available to describe the signal. Therefore, we construct each classifier sample by concatenating the transformed outputs of multiple fusion windows.

Let $L_{c}$ denote the nominal classifier sample duration in seconds, $L_{p}$ the fusion-window length in samples, and $f_{s}=3000$ samples/s the reference sampling rate used by the implementation. This value serves as a nominal conversion factor between the desired segment duration and the number of stored samples, rather than representing the physical acquisition frequency of the sensor. No resampling or interpolation was performed. The number of fusion windows contained in one classifier sample is denoted by *N* and defined as:

(1) $$N=\frac{f_{s}L_{c}}{L_{p}}.$$

Equivalently,

(2) $$L_{c}=\frac{NL_{p}}{f_{s}}.$$

Each fusion window is transformed by the fusion module into *C* components. Therefore, after transforming and concatenating the *N* fusion windows, one classifier sample has shape (N,C).

Each classifier sample uses a shorter segment of an original experiment. Its length in samples is denoted by $T_{c}$, related to its duration by:

(3) $$T_{c}=f_{s}L_{c}.$$

To construct shorter classifier samples, random starting indices $r_{i}$ were selected within each experiment. A segment contains $T_{c}=f_{s}L_{c}$ samples and must fit within the available experiment length $T_{\exp}$. The training sample count *S* is varied over 50 and 100; validation uses 100 extracted samples per experiment for both choices of *S*. The extraction boundary is:

(4) $$0\leq r_{i}<T_{\exp}-T_{c}$$

After determining the random extraction points, each selected sub-experiment was prepared for fusion and classification. Each sub-experiment has shape $\left(T_{c},9\right)$, where $T_{c}=f_{s}L_{c}$ is its length in samples, and 9 is the number of signal channels. Based on Equation (1), each sub-experiment is divided into *N* non-overlapping fusion windows, where each fusion window has shape $\left(L_{p},9\right)$ and $N=T_{c}/L_{p}$. The fusion module then transforms each fusion window into *C* components. After all *N* fusion windows are transformed and concatenated, the final fused sample has shape (N,C).

**Figure 4:**
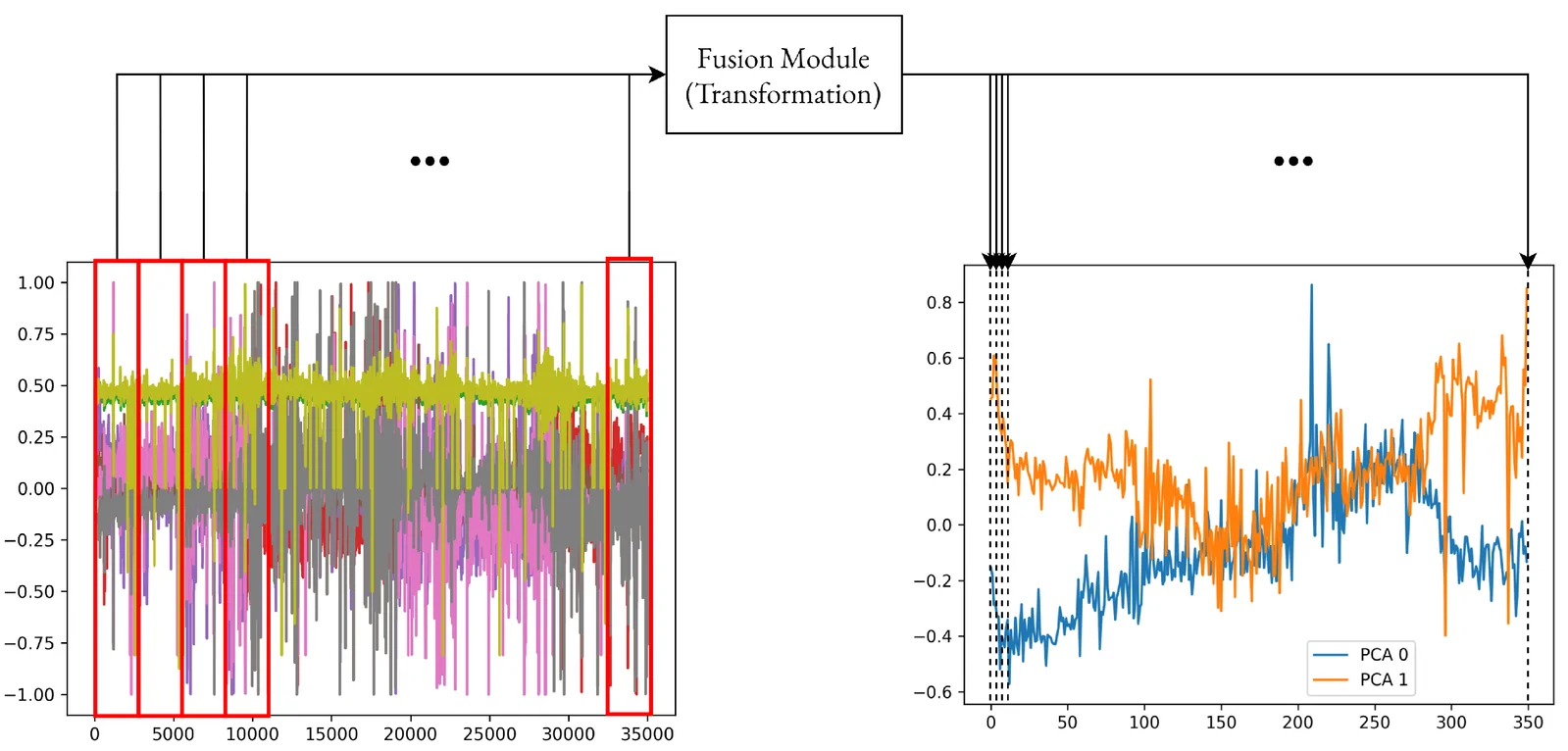
Transformation and signal fusion of an experiment. First, split the data to window frames of size $L_{p}$ and transform it using a fusion module, such as PCA. Then, concatenate the components of each transformation to reconstruct a new signal with lower dimensions.

Figure 4 illustrates this transformation process for one extracted sub-experiment. The sub-experiment is split into windows of size $L_{p}$, and each window is transformed using a fusion module such as PCA, ICA, or AVG. The transformed outputs are then concatenated to create a lower-dimensional sample that preserves the temporal structure of the original sub-experiment.

To avoid data leakage during evaluation, this process was performed separately inside each fold of the 5-fold cross-validation procedure. For each fold, the scaler was fitted only on the training experiments and then applied to both the training and validation experiments. Similarly, PCA and ICA fusion models were fitted only using the training experiments of that fold. The fitted fusion module was then used to transform both the fold-training and fold-validation sub-experiments. Therefore, the validation experiments were never used to fit either the scaler, the fusion module, or the classifier. Algorithm 1 summarizes the fold-aware construction of the fused training and validation datasets.
**Algorithm 1** Fold-Aware Fusion Dataset Construction  1:**Input:** training experiments $X_{train}$, validation experiments $X_{val}$, labels Y, fusion method *F*, fusion window length $L_{p}$, classifier duration $L_{c}$, number of outputs *C*, generated samples per experiment $S_{\max}=100$  2:Fit scaler using only $X_{train}$  3:Apply scaler to $X_{train}$ and $X_{val}$  4:$T_{c}\leftarrow f_{s}L_{c}$; require $T_{c}$ divisible by $L_{p}$  5:$N\leftarrow T_{c}/L_{p}$  6:Initialize fusion training set: Z←[]  7:**for** each experiment *X* in $X_{train}$ **do**  8:    Divide *X* into non-overlapping windows *w* of shape $\left(L_{p},9\right)$  9:    **for** each window *w* **do**10:        z←flatten(w) {*z* has shape $\left(1,9L_{p}\right)$}11:        Append *z* to *Z*12:    **end for**13:**end for**14:**if** *F* is PCA or ICA **then**15:    Fit fusion model *F* on *Z* using *C* components16:    **if** *F* is ICA **then**17:        Order components by descending contribution to reconstruction variance, estimated from *Z* only18:    **end if**19:**end if**20:Initialize fused datasets: $D_{train},D_{val}\leftarrow \left[\right]$21:**for** each split (X,D) in $\left\{\left(X_{train},D_{train}\right),\left(X_{val},D_{val}\right)\right\}$ **do**22:    **for** each experiment *X* in X **do**23:        Generate $S_{\max}$ random extraction points in $\left[0,\mathrm{length}\left(X\right)-T_{c}\right)$24:        **for** each extraction point *s* **do**25:            Extract sub-experiment $u\leftarrow X[s:s+T_{c}]$26:            Split *u* into *N* non-overlapping windows $w_{i}$ of shape $\left(L_{p},9\right)$27:            Initialize fused sample R←[]28:            **for** i=1 to *N* **do**29:                **if** *F* is PCA or ICA **then**30:                    $z_{i}\leftarrow \mathrm{flatten}\left(w_{i}\right)$31:                    $r_{i}\leftarrow F.\mathrm{transform}\left(z_{i}\right)$ {$r_{i}$ has shape (1,C)}32:                **else if** *F* is AVG **then**33:                    $r_{i}\leftarrow \mathrm{mean}\left(w_{i},\mathrm{axis}=0\right)$ {$r_{i}$ has shape (1,9)}34:                **end if**35:                Append $r_{i}$ to *R*36:            **end for**37:            Store fused sample *R* and the label of *X* in D38:        **end for**39:    **end for**40:**end for**41:**Return:** $D_{train},D_{val}$

### 2.3. Classification & Evaluation

For each seed and fold, the training experiments from four folds were combined into one training set and the remaining fold provided the validation set. Classifiers were trained on the fused training samples and evaluated on the transformed validation samples. Five-fold mean accuracies were then averaged over four seeds; fold variability was summarized across the four runs as defined below. The classifiers were:

- Support Vector Machine (SVM) [20];
- Random Forest Classifier (RF) [21];
- Multi-Layer Perceptron (MLP) [22];
- Extra Tree Classifier (ET) [23].

These models were implemented using scikit-learn 1.2.2, NumPy 1.26.1, and Python 3.10.20. In the main experiments, SVM, RF, MLP, and ET were implemented using *SVC*, *RandomForestClassifier*, *MLPClassifier*, and *ExtraTreeClassifier*, respectively. For all four classifiers, the *random state* parameter was set to the evaluation seed, and RF additionally used a non-default *n-jobs* setting for parallel execution. All remaining classifier parameters were set to their default values in scikit-learn 1.2.2. Additional fusion implementation settings are provided in Table 2.

The main goal of this project was to validate the best variables to fuse the signals and classify the textures with a shorter signal length than 12 s while keeping a high classification score.

### 2.4. The Pipeline

The pipeline evaluates different combinations of the fusion method *F*, the number of samples per training experiment *S*, the classifier model *M*, the fusion window length $L_{p}$, the classifier duration $L_{c}$, and the number of fusion components *C*. Each valid combination of these variables is referred to as a *pipeline configuration*. Figure 1 and Table 1 summarize the evaluated values. The number of fusion windows *N* is not considered an independent pipeline variable because it is determined by $L_{p}$ and $L_{c}$ (Equation (1)).

For PCA and ICA, the number of components is varied from 1 to 10. In contrast, AVG does not have a configurable number of components because it produces one average value for each of the nine input signals. Therefore, its output dimension is fixed at C=9. The number of valid pipeline configurations (P) is:

(5) $$\begin{array}{ccc} \left|P\right| & = & \left|S|\;\cdot \;|M|\;\cdot \;\right|L_{p}\left|\;\cdot \;\right|L_{c}|\left(|F_{\mathrm{PCA},\mathrm{ICA}}\left|\;\cdot \;|C|\;+\;\right|F_{\mathrm{AVG}}|\right) \end{array}$$

(6) $$\begin{array}{ccc} & = & 2\;\cdot \;4\;\cdot \;2\;\cdot \;3\left(2\;\cdot \;10+1\right) \end{array}$$

(7) $$\begin{array}{ccc} & = & 1008 \end{array}$$

Each of the 1008 pipeline configurations was evaluated using five-fold cross-validation with four seeds, giving 1008×4=4032 configuration–seed ($S_{e}$) runs and 1008×4×5 = 20,160 classifier training and validation cycles. The seed identifies a repeated evaluation, not an additional configuration in the figures.

For each configuration, accuracy was first averaged across the five validation folds within each seed. The four resulting seed-level means were then averaged with equal weight to obtain the reported accuracy. All configuration comparisons and plots use this overall mean; no individual seed was selected. Because each run contains five folds, this is also the equally weighted average of all 20 fold scores, although those scores are not independent observations.

The main results table reports mean accuracy plus or minus the root mean square (RMS) of the four within-run fold standard deviations. This summary is obtained by squaring each run’s fold standard deviation, averaging the four squared values, and taking the square root. Each run’s fold standard deviation uses a divisor of five, matching the stored summaries. The reported RMS fold standard deviation (SD) is expressed in percentage points and describes variation across validation folds; it is neither a confidence interval nor the standard deviation of all 20 fold scores. Stored seed-level means and fold standard deviations were rounded to four decimal places in accuracy units, so additional digits produced by averaging do not imply greater measurement precision.

Cross-validation was repeated using four predefined random seeds, 3141, 5926, 5358, and 9793. Each seed controlled the experiment-level fold assignment, random sub-experiment extraction, and stochastic classifier initialization. Fixing these seeds makes the runs reproducible, while repeating the evaluation with different seeds assesses sensitivity to these sources of randomness. A single seed provides only one realization of the evaluation procedure and may produce unusually favorable or unfavorable results. Evaluating each configuration across multiple seeds therefore reduces reliance on an individual run and provides a more informative description of performance stability.

For each seed, the experiments were shuffled and divided into five folds at the experiment level using that seed. The same seeded partition was used across configurations within that repeat. For each fold, one group of experiments was used for validation, and the remaining four groups were used for training. All sub-experiments extracted from a validation experiment remained in the validation set, thereby preventing samples from the same experiment from appearing in both training and validation.

For each fold, the min–max scaler (normalization) was fitted using only the fold-training experiments. The fitted scaler was then applied to both the training and validation experiments. Similarly, PCA and ICA were fitted only on fusion windows obtained from the scaled fold-training experiments, while AVG does not require a fitting step. The fitted fusion module was subsequently used to transform both training and validation sub-experiments, as described in Algorithm 1.

To reduce repeated computation, the pipeline generated the maximum required number of sub-experiments and fusion components and then selected subsets for smaller configurations. For a given fold, fusion method, $L_{p}$, and $L_{c}$, the generated arrays have the following shapes:

(8) $$\begin{array}{ccc} X_{\mathrm{split}}.\mathrm{shape} & = & \left(\left|\mathrm{textures}|,\right|{\mathrm{experiments}}_{\mathrm{split}}|,S_{\max},N,C_{\max}^{F}\right), \end{array}$$

(9) $$\begin{array}{ccc} Y_{\mathrm{split}}.\mathrm{shape} & = & \left(\left|\mathrm{textures}|,\right|{\mathrm{experiments}}_{\mathrm{split}}|,S_{\max}\right), \end{array}$$

where $C_{\max}^{F}=10$ for PCA and ICA and $C_{\max}^{F}=9$ for AVG. The value of *N* is fixed for each $\left(L_{p},L_{c}\right)$ combination and is therefore not a dimension over which configurations are selected.

Unlike PCA, ICA does not naturally order its components by importance. Therefore, after fitting the 10-component ICA model within each cross-validation fold, we ranked its components by descending contribution to reconstruction variance. This contribution accounts for both the variability of each recovered source and the strength of its contribution to reconstructing the scaled input signals. The ranking was estimated exclusively from the fold-training windows and applied consistently to training and validation data. Configurations with fewer components retained the first *C* components from this ordered basis. Thus, smaller configurations represent subsets of a fixed 10-component ICA model rather than separately fitted ICA models.

For a configuration containing $S_{i}$ training samples per experiment and $C_{i}$ components, the first $S_{i}$ samples and first $C_{i}$ components are selected from the maximum-size training array. Each fused sample, originally having shape $\left(N,C_{i}\right)$, is flattened into a feature vector of length $NC_{i}$. Consequently, the classifier training data have the shape:

(10) $$X_{\mathrm{train}}^{\left(i\right)}.\mathrm{shape}=\left(\left|\mathrm{textures}|\right|{\mathrm{experiments}}_{\mathrm{train}}|S_{i},\;NC_{i}\right),$$

and the corresponding label vector has length $\left|\mathrm{textures}|\right|{\mathrm{experiments}}_{\mathrm{train}}\left|S_{i}\right|$. Algorithm 2 summarizes the complete training and five-fold evaluation procedure.
**Algorithm 2** Repeated Five-Fold Cross-Validation Evaluation Pipeline  1:**Input:** experiments, labels, fusion methods, classifiers, $L_{p}$, $L_{c}$, S={50,100}, seeds $S_{e}$  2:**for** each seed $s\in S_{e}$ **do**  3:    Initialize extraction RNG and classifier random states with *s*  4:    Shuffle and split experiments into five folds using *s*  5:    **for** each fusion method *F* and fusion-window length $L_{p}$ **do**  6:        Set $C_{F}=\left\{1,\ldots ,10\right\}$ for PCA/ICA, or {9} for AVG  7:        **for** each fold k=1,…,5 **do**  8:            Use fold *k* for validation and the remaining folds for training  9:            Fit scaler and fusion model on training experiments only, retaining $\max C_{F}$ outputs10:            **if** *F* is ICA **then**11:               Rank components by descending contribution to reconstruction variance, estimated from training data only12:               Use the same component ordering for training and validation data13:            **end if**14:            **for** each duration $L_{c}$ **do**15:               Extract and transform 100 samples per experiment in both splits, using Algorithm 1’s construction and reusing the fitted transformations16:               **for** each S∈S, $C\in C_{F}$, and classifier *M* **do**17:                  Select the first *S* training samples and *C* outputs; retain all 100 validation samples with the same *C* outputs (For ICA, the first *C* components are selected from variance ordering)18:                  Flatten samples to NC features; train *M* and record validation accuracy19:               **end for**20:            **end for**21:        **end for**22:    **end for**23:    Calculate each configuration’s five-fold mean accuracy and fold SD24:**end for**25:Average each configuration’s four seed-level means and calculate the RMS of its four-fold SDs26:Select maximum-mean configurations and calculate the mean-accuracy Pareto frontiers

For the trial-level confusion matrix presented in Section 3, we selected PCA–RF configurations and evaluated using five-fold experiment-level cross-validation with seed 3141. Scaling and PCA were fitted exclusively on the training trials within each fold. For each nominal segment duration, one prediction per held-out trial was obtained from its first predefined randomly extracted validation segment, without aggregation across segments. These out-of-fold predictions were pooled across the five folds, yielding 1200 trial-level predictions per duration, with 100 trials per texture and each trial counted once. Confusion-matrix counts, class-wise precision and recall, and overall accuracy were calculated from these pooled predictions. These single-seed, one-segment-per-trial results are distinct from the four-seed, segment-level configuration-search estimates that are presented and discussed in the last two sections (Section 3 and Section 4).

### 2.5. Baseline Comparisons and Sensor Ablation

In order to have a baseline method, the random forest approach of Lima et al. [6] was adapted to individual MARG channels using 3, 6, and 9 s segments from the 30 mm/s subset. A stratified trial-level 80/20 split was used following the same methodology as the previous authors. One random segment per trial was shared across channel comparisons, with temporal-feature min–max scaling fitted only on training segments. RF used 500 trees, maximum depth 30, Gini impurity, bootstrap sampling, square-root feature sampling, and seed 3141.

Raw concatenation of all nine channels was compared with PCA–RF using identical holdout trials, segment locations, and RF settings. Both used training-only channel-wise min–max scaling. PCA used non-overlapping flattened windows, randomized SVD, 10 fitted components, 7 power iterations, 10 oversamples, no whitening, and seed 3141. Accuracy used one prediction per test trial.

To better understand how each channel contributes to the results, sensor ablation was performed. The ablation methodology retained each sensor group individually, each pair, and all MARG channels, using identical five-fold trial partitions with seed 3141. Scaling and PCA were refitted on complete training trials for each subset, retaining the preceding window/component settings. RF used 100 trees with unrestricted depth and 100 random training segments per trial, while other RF settings were unchanged. Validation used one predetermined segment per trial, reporting mean accuracy and sample fold SD. Approximate channel importance was allocated to PCA features via mean deviation index (MDI) through squared PCA loadings and was summarized across folds.

## 3. Results

All results in this section use configuration means across four seeds, with five-fold cross-validation within each seed. Table 3 selects the configuration with the highest unrounded four-seed mean for each method and duration, where exact ties favor fewer features. Throughout this section, the best observed CV estimate denotes the highest four-seed mean within the corresponding configuration group. Configuration selection and performance reporting use the same CV results, so these estimates may be optimistic.

At $L_{c}=3$ s, the highest means are 99.41% for AVG, 99.09% for PCA, and 98.92% for ICA. PCA and ICA tie their mean scores at 6 s (99.92%), while PCA wins at 9 s (99.99%). The table reports mean accuracy plus or minus the RMS of the four within-run fold standard deviations. These descriptive maxima are selected and evaluated using the same CV estimates and do not establish statistically significant differences among methods or unbiased performance after selection.

The configurations with the highest observed CV means at 3 s contain 162, 162, and 90 features for AVG, PCA, and ICA, respectively. At 6 s, PCA uses 96 features, compared with 108 for AVG and 144 for ICA. At 9 s, the corresponding lengths are 126 for PCA, 162 for AVG, and 162 for ICA. These maxima should be distinguished from compact operating points selected by considering accuracy and feature count jointly.

**Figure 5:**
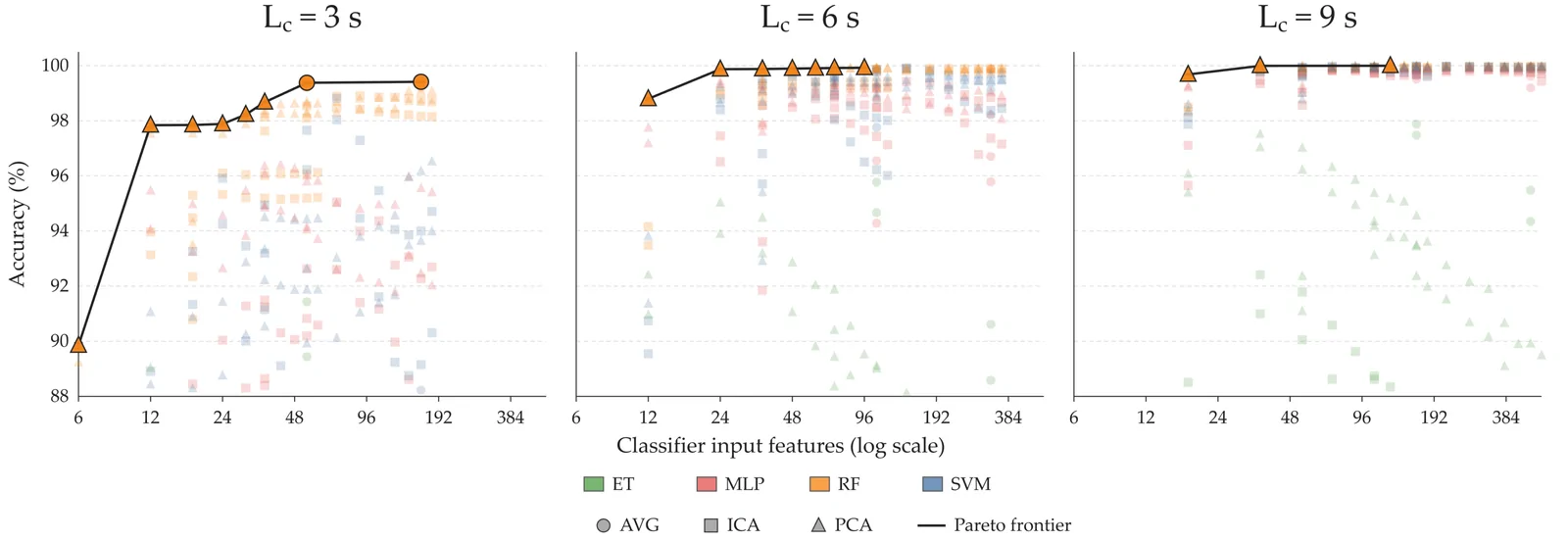
Mean cross-validation accuracy versus classifier input length for $L_{c}=3,6,$ and 9 s. Each point represents a fixed configuration’s mean across five-fold CV runs. Large outlined markers connected by a solid line identify non-dominated configurations: no other configuration has at least as high a mean accuracy and no more features, with a strict improvement in at least one objective. Configurations with means below 88% are omitted visually, but the frontier uses the complete grid.

Figure 5 shows this trade-off over the complete configuration grid. At 3 s, PCA–RF achieves 97.84% with 12 features, while AVG–RF reaches 99.38% with 54 features and 99.41% with 162 features. At 6 s, the frontier contains only PCA–RF configurations, from 98.79% with 12 features to 99.92% with 96 features; the 72-feature point achieves 99.92%. At 9 s, PCA–RF reaches 99.68% with 18 features, 99.99% with 36 features, and the same displayed accuracy after rounding with 126 features. RF supplies every displayed frontier configuration. The 36-feature and 126-feature PCA–RF configurations have the same displayed accuracy after rounding. The 126-feature configuration has the higher unrounded CV mean, but this difference does not establish a meaningful performance improvement. The 36-feature configuration therefore offers a compact alternative.

**Figure 6:**
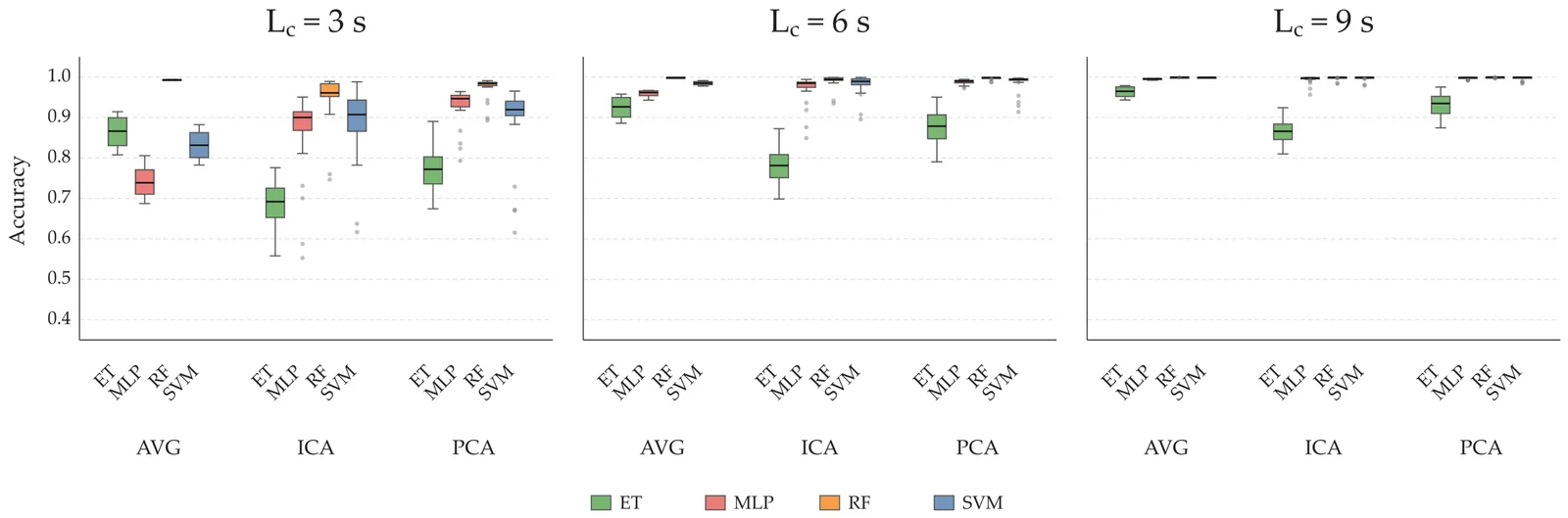
Distributions of configuration-mean CV accuracy for AVG, ICA, and PCA, separated by classifier and duration $L_{c}$. Each observation averages the CV means for one fixed configuration.

Increasing $L_{c}$ raises the median accuracy for every fusion-method/classifier group in Figure 6. The boxplot shows that RF has the highest median in all nine method/duration groups, although the distributions also reflect the chosen configuration grid. The distributions describe sensitivity across configurations, not independent repeated datasets. AVG has four configurations per box, whereas PCA and ICA have 40; these different grids must be considered when comparing their distributions. In a preliminary comparison of PCA and reconstruction-variance-ordered ICA, PCA has higher mean CV accuracy in 408 of 480 matched configurations with identical $M,S,L_{p},L_{c},$ and *C*, while ICA has higher accuracy in 55 configurations and 17 are tied. Mean accuracy over these matched grids is 94.68% for PCA and 90.09% for ordered ICA. The counts are descriptive and are not independent trials for a significance test.

The component sweeps below maximize the four-seed configuration mean over classifier and training sample count for each fixed $\left(F,L_{c},L_{p},C\right)$. They do not select the best individual seed.

For PCA with $L_{p}=500$, increasing *C* from 1 to 2 gives the following best configuration means: at 3 s, 94.33% to 98.67%, with a sweep maximum of 99.09% at C=9; at 6 s, 99.52% to 99.90%, with a sweep maximum of 99.90% at C=2,10; at 9 s, 99.92% to 99.99%, with a sweep maximum of 99.99% at C=2,3,4.

For PCA with $L_{p}=1500$, increasing *C* from 1 to 2 gives the following best configuration means: at 3 s, 89.85% to 97.84%, with a sweep maximum of 98.62% at C=8,10; at 6 s, 98.78% to 99.87%, with a sweep maximum of 99.92% at C=8; at 9 s, 99.68% to 99.99%, with a sweep maximum of 99.99% at C=7,8,9.

All three accuracy-optimal 3 s configurations use $L_{p}=500$. At 6 and 9 s, the selected AVG and PCA configurations use $L_{p}=1500$. RF provides seven of the nine accuracy-optimal configurations in Table 3, while SVM provides the selected ICA configurations at 6 and 9 s. These observations motivate the discussion of averaging at short durations, sensitivity to the retained component count, and the trade-off between exploration time and feature length.

**Figure 7:**
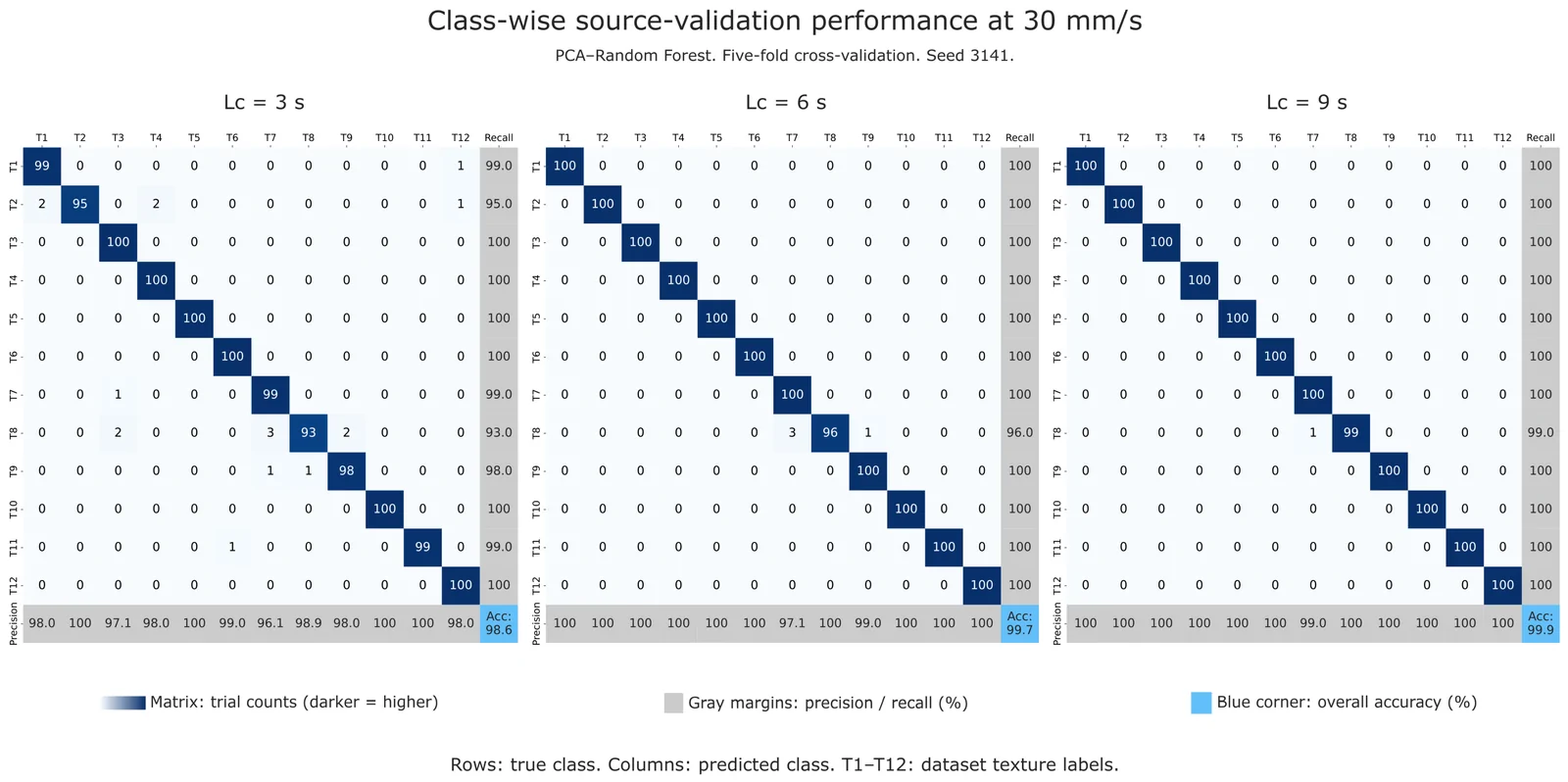
Class-wise PCA–RF confusion matrices at 30 mm/s for $L_{c}=3$, 6, and 9 s using five-fold cross-validation (seed 3141). Rows indicate true classes and columns predicted classes (T1–T12). Cells show trial counts; gray margins report precision and recall (%), and blue corners show overall accuracy (%).

Figure 7 presents the best model configuration with class-wise source-validation performance of PCA–RF for $L_{c}=3$, 6, and 9 s, using five-fold cross-validation with seed 3141. Overall accuracy increases from 98.6% at 3 s to 99.7% at 6 s and 99.9% at 9 s. T8 has the lowest recall at each duration, improving from 93% to 96% and 99%, respectively; at 6 and 9 s, all remaining classes achieve 100% recall. These single-seed results illustrate the distribution of classification errors across textures.

To assess the contribution of the input representation and sensor selection, and since RF was the best model on average, we use it to run additional experiments compared with individual raw channels, raw multichannel concatenation, and PCA-based representations. Sensor-group ablations further evaluated whether high accuracy was retained with fewer sensing modalities. The holdout comparisons and five-fold ablations are reported separately under their respective evaluation protocols.

Table 4 reports the single-channel raw-signal RF baselines under the trial-level holdout protocol. The highest observed accuracy increased from 41.67% at 3 s to 76.67% at 9 s. Magnetometer Z provided the highest accuracy at 3 and 6 s, whereas accelerometer Z performed best at 9 s. These results describe individual raw channels without PCA or multichannel concatenation.

Table 5 compares raw concatenation of all nine channels with PCA–RF using identical holdout trials, segments, and RF settings. PCA–RF increased accuracy by 29.17, 39.58, and 21.25 percentage points at 3, 6, and 9 s, respectively, while reducing the input to 162, 96, and 126 features. These holdout estimates are separate from the repeated-CV configuration-search results reported above.

This holdout comparison used one training segment per trial, whereas the selected models in Table 3 used 100, and the PCA fitting and evaluation protocols also differed. These differences may contribute to the accuracy gap, so the gains reported here describe the matched holdout setting rather than establishing an advantage under the main CV training regime.

**Figure 8:**
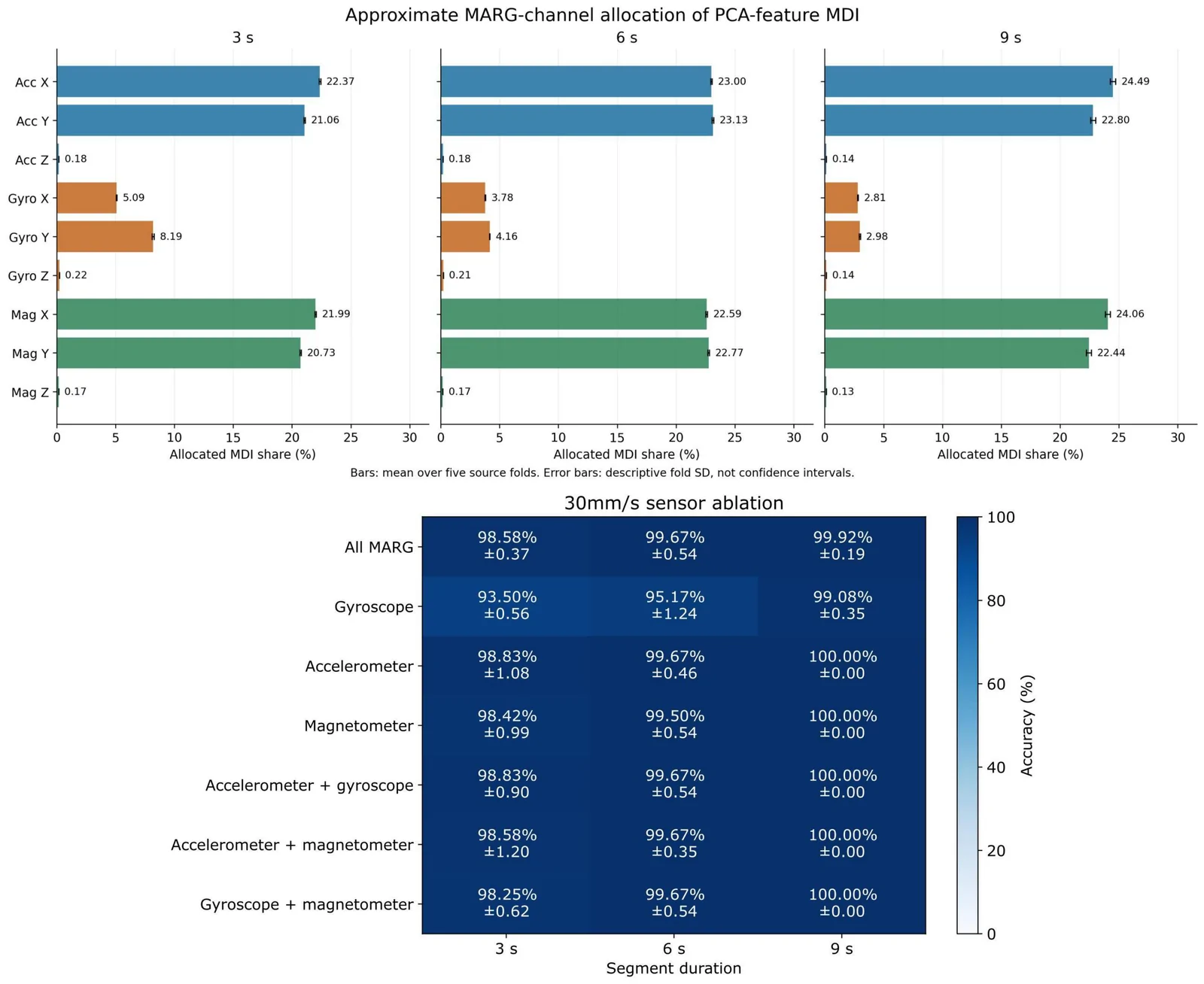
Approximate channel attribution and sensor-group ablation at 30 mm/s. Upper panels show PCA-feature MDI allocated to channels through squared PCA loadings, averaged across five folds; error bars indicate sample fold SD. The lower panel reports mean accuracy and sample fold SD for the retained sensor groups, with scaling, PCA, and RF refitted for each subset. All rows, including all MARG, use the matched seed-3141 five-fold protocol and one predetermined validation segment per trial. Accuracy is expressed in percent and SD in percentage points; SD bars are descriptive, not confidence intervals.

Figure 8 presents the sensor-group ablation under the separate five-fold protocol. Accelerometer-only accuracy was 98.83%, 99.67%, and 100.00% at 3, 6, and 9 s, respectively; magnetometer-only accuracy was 98.42%, 99.50%, and 100.00%. Gyroscope-only accuracy was lower, ranging from 93.50% to 99.08%. The matched all-MARG reference achieved 98.58%, 99.67%, and 99.92%. Thus, retaining all three sensor groups did not consistently increase observed accuracy. The approximate MDI allocation predominantly assigned importance to the accelerometer and magnetometer X and Y channels; this describes the fitted PCA–RF representations rather than the effect of sensor removal.

## 4. Discussion

The objective was to assess whether nine MARG channels can provide compact texture representations from shortened portions of the original tactile recordings. Under the evaluated protocol, segments containing 9000 stored rows, corresponding to a nominal duration of 3 s under the implementation’s reference conversion of 3000 rows/s, produced the best observed CV estimates of 99.41%, 99.09%, and 98.92% for AVG, PCA, and ICA, respectively. Because this reference conversion does not establish exact physical elapsed time, these results should not be interpreted as demonstrating a verified 75% reduction in interaction duration relative to the original 12 s recordings. These estimates summarize the configuration search and may be optimistic because the same CV results were used for selection and reporting. Previous work on this dataset reported approximately 100% accuracy using the complete 12 s interaction and a single signal [6]. Differences in evaluation protocols prevent a direct performance-equivalence claim. The present results nevertheless demonstrate high observed classification accuracy using shortened portions of the recordings under the evaluated acquisition condition.

AVG performs strongly at $L_{c}=3$ s. It averages each channel within a fusion window, retaining nine temporal summaries for RF classification. Its effectiveness suggests that channel-level summaries contain discriminative information under the evaluated trajectory, although the contribution of fluctuation suppression was not isolated experimentally. At 3 s, AVG–RF reaches 99.38% with 54 features, whereas PCA–RF achieves 97.84% with 12 features. The AVG configuration with the highest observed CV mean uses 162 features, with an estimate of 99.41%.

Among the learned methods, PCA has a higher four-seed mean CV accuracy than ICA, where PCA leads in 408/480, and ICA leads in 55 and 17 ties. The 6 s and 9 s frontiers contain only PCA–RF configurations. At 9 s, PCA–RF achieves 99.68% with 18 features and a maximum accuracy of 99.99% with 126 features. The small percentage-point gain provides little basis for preferring the larger representation without independent evaluation. For comparison, the 162-feature ICA–SVM configuration averages 99.98% across the four seeds and is dominated by the 36-feature PCA–RF point, not an alternative Pareto optimum.

PCA’s observed advantage may relate to correlations among channels generated by the same contact and motion, which PCA represents through ordered orthogonal components. ICA seeks statistically independent latent sources. However, the approximate PCA-loading attribution does not establish source independence or explain the PCA–ICA performance difference, so these explanations remain hypotheses. The seed comparisons also indicate method-dependent sensitivity: repeated evaluations assess the combined effects of fold assignment, extraction, and classifier randomness. The RMS fold SD in the main table summarizes variability across validation folds within the four runs. It does not establish statistical significance or equivalence between methods.

The additional baselines clarify the contribution of PCA-based representation. In the matched holdout comparison, PCA–RF improved accuracy over raw nine-channel concatenation by 29.17, 39.58, and 21.25 percentage points at 3, 6, and 9 s, respectively, while substantially reducing feature count (Table 5). These results support the usefulness of PCA relative to raw concatenation under the tested one-segment-per-trial training regime and RF settings. They do not isolate the benefit of adding sensors, establish superiority over AVG, or provide an evaluation independent of the earlier configuration selection.

The sensor-group ablations indicate substantial redundancy under the evaluated acquisition condition (Figure 8). Accelerometer-only and magnetometer-only PCA–RF retained high accuracy, while using all MARG channels did not consistently improve observed performance. At 9 s, their 100% accuracies correspond to zero errors, compared with one error for the matched all-MARG model (99.92%). This difference does not establish superiority of the reduced sensor sets. The approximate MDI allocation provides descriptive attribution within the fitted PCA–RF models, rather than evidence of sensor necessity. Because the ablations use one seed and one validation segment per trial, their scores should be interpreted separately from the four-seed, segment-based estimates in Table 3.

The study remains limited to one dataset, sensing system, controlled texture set, exploration velocity of 30 mm/s, and fixed exploration trajectory. Although the configuration search uses four seeds, all repeats reuse the same original trials and do not constitute independent datasets. Generalization to other velocities has not been established. Preliminary cross-velocity transfer experiments showed poor performance, but a complete controlled evaluation was not finalized, and quantitative transfer results are therefore not reported. A future speed-aware recognition system could use commanded exploration velocity or an estimate of actual sliding velocity to condition the classifier or normalize its inputs. Such an approach would require velocity information during deployment, whereas a representation intended to generalize without that information would need explicit validation across velocities. Neither approach was evaluated here.

Changes in exploration velocity may alter the temporal and amplitude characteristics of the recorded signals; however, their contribution to the observed transfer difficulty was not isolated. The present conclusions are consequently restricted to the evaluated acquisition condition. The barometer was excluded, so the findings also do not establish whether adding pressure measurements would improve classification.

Experiment-level splitting prevents segments from the same trial from appearing in both training and validation within a fold. Nevertheless, overlapping segments from one trial are correlated, and segment-level accuracy should not be interpreted as evidence from independent experimental replications. Trial-clustered uncertainty estimates were not calculated for the configuration search, which is a limitation of this study. The reported fold standard deviations are descriptive measures of variability across folds and do not quantify uncertainty while accounting for dependence among segments from the same trial. Individual fold scores and experiment-level predictions for all configurations were not retained in the saved result summaries, preventing a complete retrospective paired analysis and trial-clustered uncertainty assessment of the configuration search. Configurations were also selected using the same CV means used to report their performance; nested CV or an untouched experiment-level test set is needed to assess performance after selection.

The segment labels of 3, 6, and 9 s are nominal conversions from stored-row counts rather than verified elapsed durations for every evaluated segment. The results therefore do not establish an exact percentage reduction in physical interaction time. Latency, memory use, and energy consumption were not measured, so shorter feature vectors do not by themselves establish real-time or resource-efficiency benefits.

Taken together, these findings connect the fusion analysis to robotic tactile perception by showing how compact representations of the recorded exploration signals support texture discrimination. The strong performance of AVG at 3 s suggests that channel-wise temporal means, concatenated across successive windows, retain substantial discriminative information under the evaluated exploration protocol. PCA instead represents joint variation across channels and time within each fusion window, allowing accurate classification with compact feature vectors. For robotic texture recognition, these results motivate evaluating both simple temporal summaries for short observations and learned compact representations when longer observations are acceptable. These interpretations concern the classification information retained by the representations; they do not establish that individual components correspond to specific physical texture properties.

Future work should evaluate other trajectories, speeds, directions, contact forces, textures, and robotic platforms, as well as durations below 3 s. Retaining fold assignments, predictions, convergence diagnostics, and separate split, extraction, and model seeds would allow a more detailed robustness assessment. Runtime measurements on the target robot are also needed. The present results support AVG for high observed accuracy at short durations and PCA for compact representations at longer durations, subject to these evaluation limits.

Aref Sayareh initially led the core work at Memorial University of Newfoundland during his master’s degree, after which Frederico Luiz Martins de Sousa and Mateus Nazario Coelho took over and expanded his research contributions through data curation, software development, and manuscript writing. Mateus Coelho Silva from Universidade Federal do ABC provides external co-supervision, validation, and review, while Dr. Amilcar Soares Junior, who was previously associated with both Memorial University of Newfoundland and Linnaeus University, now continues his international collaboration from the University of Bristol. Dr. Thiago Eustaquio Alves de Oliveira from Lakehead University and I (who currently hold a joint affiliation with Memorial University of Newfoundland and Linnaeus University) are jointly leading this research project and providing primary oversight, project administration, and conceptual direction for the entire team. Please let me know if you have further questions.

## Figures and Tables

**Table 1:** Pipeline configuration variables and their values. Seeds are four consecutive four-digit blocks from the digits of π with the decimal separator omitted, starting with the integer digit. The seed identifies a repeated evaluation, not a distinct pipeline configuration.

Variable	Description	Symbol	Values
Fusion method	Method used to transform each fusion window into a lower-dimensional representation	*F*	PCA, ICA, AVG
Samples per training experiment	Number of extracted sub-experiments used per training experiment	*S*	50, 100
Classifier model	Machine learning model used to classify the extracted samples	*M*	MLP, ET, RF, SVM
Fusion window size	Length of each small window passed to the fusion method	$L_{p}$	500, 1500 samples
Sample window size	Duration of each extracted sub-experiment used as one classifier sample	$L_{c}$	3, 6, 9 s
Components	Number of fused components retained from the fusion method	*C*	1, 2, 3, 4, 5, 6, 7, 8, 9, 10
Fusion windows per sample	Number of fusion windows contained in one sample window, computed as $N=f_{s}L_{c}/L_{p}$, with $f_{s}=3000$ samples/s (nominal reference rate)	*N*	6, 12, 18, 36, 54
Random seeds	Controls fold assignment, extraction, and classifier randomness for reproducible repeated runs	$S_{e}$	3141, 5926, 5358, 9793

**Table 2:** Additional fusion implementation settings. The software environment used Python 3.10.20, NumPy 1.26.1, and scikit-learn 1.2.2. The configuration variables reported in Table 1 are omitted here.

Method	Parameter	Setting
PCA	SVD solver	Randomized
	Power iterations	7
	Oversampling	10
	Whitening	Disabled
	Random state	0
AVG	Fitted parameters	None; mean of the $L_{p}$ rows of each window, per channel
ICA	Algorithm	FastICA, parallel
	Whitening	arbitrary-variance, SVD solver
	Nonlinearity	logcosh, α=1.0
	Maximum iterations	200
	Convergence tolerance	$10^{-4}$
	Initial unmixing matrix	Random, drawn from the random state
	Random state	0
	Component ordering	Decreasing training-set reconstruction variance

**Table 3:** These are the best observed CV estimates selected using the same results used for reporting. Configuration with the highest mean cross-validation accuracy across four seeds (3141, 5926, 5358, and 9793), selected separately for each fusion method and touch-exploration duration. Mean accuracy averages the four seed-level five-fold CV mean accuracies. Values are mean accuracy ± RMS fold SD, expressed in percentage points. Exact mean ties favor fewer input features. For PCA and ICA, *C* is the retained component count, and for AVG, C=9 represents one temporal average per input channel.

$L_{c}$ (s)	Method	Classifier	*S*	$L_{p}$	*C*	Input Features	Accuracy (%) Mean ± RMS Fold SD
3	AVG	RF	100	500	9	162	99.41±0.20
3	PCA	RF	100	500	9	162	99.09±0.20
3	ICA	RF	100	500	5	90	98.92±0.11
6	AVG	RF	100	1500	9	108	99.90±0.11
6	PCA	RF	100	1500	8	96	99.92±0.05
6	ICA	SVM	100	500	4	144	99.92±0.09
9	AVG	RF	100	1500	9	162	99.97±0.06
9	PCA	RF	100	1500	7	126	99.99±0.01
9	ICA	SVM	100	500	3	162	99.98±0.02

Bold indicates the largest mean within each duration.

**Table 4:** Single-channel raw-signal RF accuracy on the stratified trial-level holdout. Each duration uses one segment per trial, with identical segment locations across channels. Bold values indicate the highest observed accuracy within each duration.

Channel	3 s (%)	6 s (%)	9 s (%)
Accelerometer X	33.33	45.00	58.75
Accelerometer Y	36.25	37.92	61.67
Accelerometer Z	40.00	51.25	**76.67**
Gyroscope X	20.42	25.83	38.75
Gyroscope Y	20.00	24.17	40.00
Gyroscope Z	28.75	33.33	53.33
Magnetometer X	33.75	48.33	63.33
Magnetometer Y	34.17	39.17	64.58
Magnetometer Z	**41.67**	**52.92**	75.83

**Table 5:** Matched holdout comparison of raw nine-channel concatenation and PCA–RF. Gain is the PCA–RF accuracy minus raw RF accuracy, calculated before rounding.

Duration	Raw RF	PCA–RF	Gain	Raw	PCA
(s)	(%)	(%)	(pp)	Features	Features
3	47.50	**76.67**	+29.17	81,000	162
6	57.08	**96.67**	+39.58	162,000	96
9	78.33	**99.58**	+21.25	243,000	126

Bold indicates the largest mean within each duration.

## Data Availability

The data presented in this study are publicly available in Mendeley Data at https://doi.org/10.17632/n666tk4mw9.1.
